# Unusual presentation of lepidic adenocarcinoma in a healthy female

**DOI:** 10.1186/s12890-022-01969-1

**Published:** 2022-05-16

**Authors:** Zaheer Akhtar, Leah Laageide, Julian Robles, Christopher Winters, Geoffrey C. Wall, James Mallen, Zeeshan Jawa

**Affiliations:** 1 PGY3 Internal Medicine Resident, Department of Medical Education, UnityPoint Health, Des Moines, IA USA; 2 Department of Medicine, UnityPoint Health, Des Moines, IA USA; 3 Department of Surgery, UnityPoint Health, Des Moines, IA USA; 4 Drake College of Pharmacy and Health Sciences, Des Moines, IA USA; 5 Department of Pulmonology, The Iowa Clinic and UnityPoint Health, Des Moines, IA USA; 6 John Stoddard and Mission Cancer Center, Des Moines, IA USA

**Keywords:** Non small cell lung cancer, Adenocarcinoma, Lepidic pattern, Bronchioloalveolar carcinoma, Micronodular infiltrates, Case report

## Abstract

**Background:**

Lepidic adenocarcinoma represents a histologic pattern of non-small cell lung cancer that characteristically arises in the lung periphery with tracking alongside pre-existing alveolar walls. Noninvasive and invasive variants of lepidic adenocarcinoma are dependent on parenchymal destruction, vascular, or pleural invasion. The lepidic-predominant lung malignancies are collectively recognized as slow growing with rare metastasis and excellent prognosis. The World Health Organization classification of lung malignancies depends on molecular and histopathological findings. CT findings most commonly include ground-glass characteristics, commonly mistaken for inflammatory or infectious etiology. These tumors are generally surgically resectable and associated with better survival given infrequent nodal and extrathoracic involvement. Rarely these tumors present with diffuse pneumonic-type involvement associated with worse outcomes despite lack of nodal and distant metastases.

**Case presentation:**

A 63-year-old Caucasian athletic immunocompetent female presented with 2 months of progressive shortness of breath, fatigue, loss of appetite and 15 pound weight loss. History was only notable for well controlled essential hypertension and hypothyroidism. Contrast computed tomography angiogram and positron emission tomography revealed diffuse hypermetabolic interstitial and airspace abnormalities of the lungs without lymphadenopathy (or distant involvement) in addition to right hydropneumothorax and left pleural effusion. Baseline laboratory testing was unremarkable, and extensive bacterial and fungal testing returned negative. Bronchoscopy and video-assisted thoracoscopic surgery was subsequently performed with pleural fluid cytology, lung and pleural biopsies returning positive for lepidic adenocarcinoma with 2% programmed death ligand 1 expression and genomic testing positive for PTEN gene deletion. Prior to treatment, the patient perished on day 15 of admission.

**Conclusion:**

We present a rare case of lepidic predominant adenocarcinoma with extensive bilateral aerogenous spread in the context of no lymphovascular invasion in a healthy, low risk patient. This case presentation may add to the body of knowledge regarding the different behavior patterns of lepidic predominant adenocarcinomas.

## Background

Lung cancers are twice as common as intra-abdominal cancers and therefore compromise the largest class of malignancies and cancer-related deaths worldwide [1]. Lung cancers are divided into two types: non-small cell lung cancer (NSCLC; approximately 85% of lung malignancies), and small cell lung cancer (SCLC) [2]. Among the subtypes of lung cancer, adenocarcinoma (a NSCLC) accounts for nearly 50% of cases generally associated with mutations in the kirsten rat sarcoma gene (KRAS), anaplastic lymphoma kinase gene (ALK), or epidermal growth factor receptor (EGFR) [3]. Primary group classification (preinvasive, minimally invasive and invasive adenocarcinomas) is dependent on growth pattern predominance of the neoplastic cells (defined as a percentage and recorded in 5% increments), size of lesion, or invasion [4]. All these groups can present in a purely lepidic or predominant lepidic-pattern (defined as columnar or cuboidal cell proliferation that characteristically tracks alongside intact alveolar walls without stromal or lymphovascular invasion). Prognostically, 70–90% of adenocarcinomas are diagnosed as invasive (defined as greater than 3.0 cm in size and/or greater than 0.5 cm of pleural, lymphatic, or vascular invasion), of which the lepidic pattern is considered favorable, while micropapillary or solid pattern are unfavorable [5–7]. Lepidic predominant adenocarcinoma (LPA), formerly called non-mucinous bronchoalveolar carcinoma, has CT findings of both ground glass and solid opacities which corresponds to the invasive component [8]. Histologically, LPA type cancers show non-mucinous, type II pneumocyte and/or Clara cell proliferation along alveolar walls with greater than 3 cm in size as well as vessel or pleural involvement [9–11]. In contrast to most adenocarcinomas, LPA primarily affects women and is rare with an incidence of 4%. Aerogenous spread of LPA is exponentially rarer. The most common presenting symptoms of LPA are weight loss (35–45%), dyspnea (37–45%), and weakness (34%) [1, 12]. We present an unusual case of this presentation in an immunocompetent 63-year-old female.

## Case presentation

A 63-year-old, Caucasian and thin (BMI 17.06 kg/m^3^) female presented to the emergency department for 2 months of progressively worsening shortness of breath, loss of appetite, and weight loss (15 pounds). She also reported worsening fatigue limiting her ability to exercise, and also dyspnea with mild-moderate exertion. The patient denied any additional constitutional symptoms. Medical history was notable for hypothyroidism secondary to Hashimoto thyroiditis and well-controlled essential hypertension. Patient was up to date on screening tests including a negative colonoscopy 3 months prior. Social history included intermittent social tobacco use (defined as using 1–2 cigarettes in a setting, while at parties or social gatherings over a 5-year period in her early 30 s). Additional pertinent history included occasional alcohol use, no illicit drug use, and a healthy lifestyle with focus on healthy eating and outdoor bicycling. Family history was only notable for type II diabetes mellitus and obesity.

**Fig. 1:**
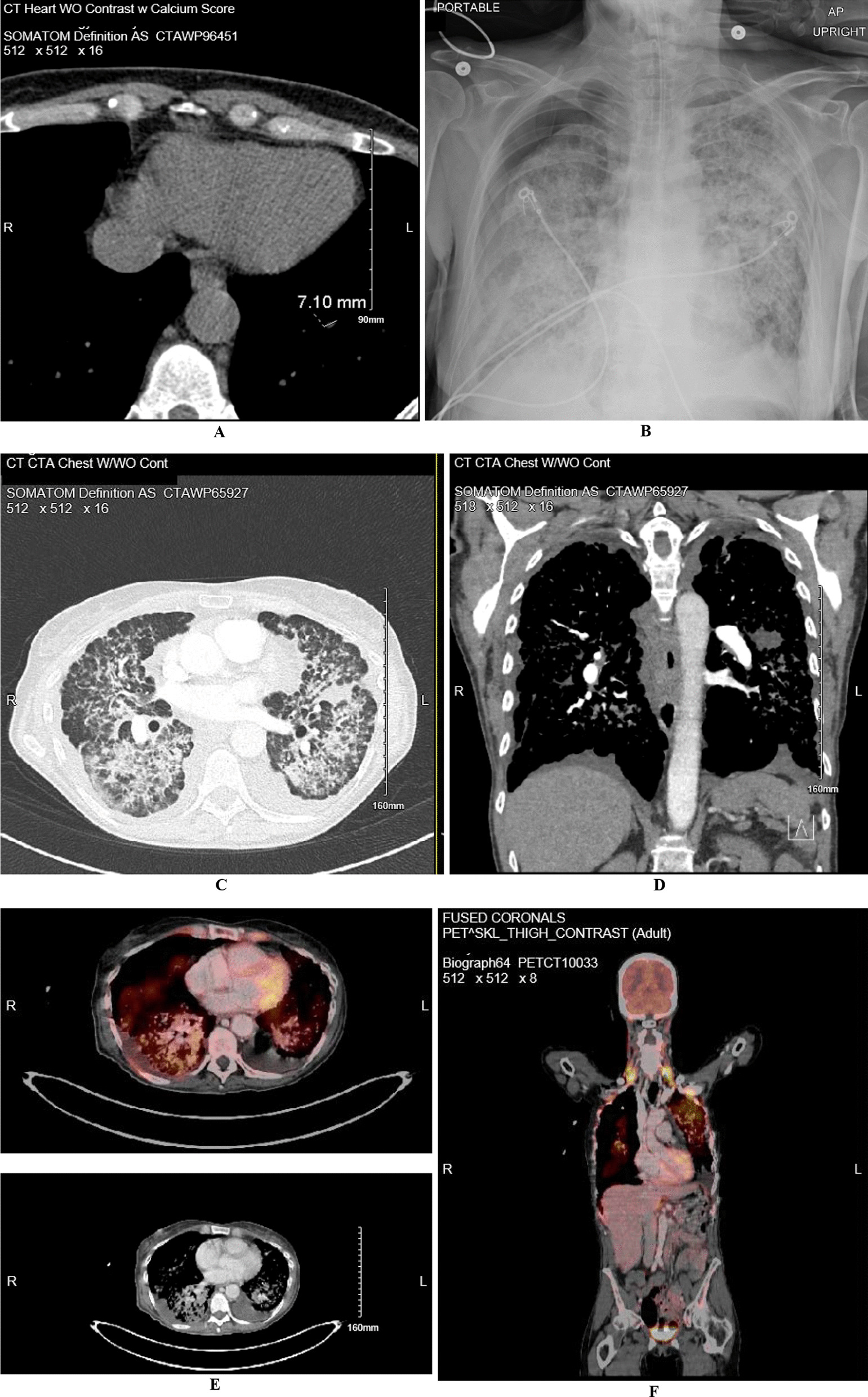
**A, B** Computed tomography (CT) without contrast with calcium score performed 3 year prior to admission for evaluation of coronary artery disease showing a score of zero in all branches and a 7 mm nodule in the left lower lobe. **B** Day of admission 1-view chest X-ray (CXR) showing a right pneumothorax with a 3.2 cm pleural separation, prominent diffuse bilateral interstitial alveolar opacities. **C**, **D** CT chest with and without contrast showing diffuse nodular opacities throughout the lungs with areas of coalescence, in addition to small bilateral pleural effusions. **E** Positron emission tomography (PET) showing diffuse hypermetabolic interstitial and airspace abnormality of the lungs without lymphadenopathy (or distant lesions), in addition to right hydropneumothorax and left pleural effusion

One week before admission, the patient underwent primary care directed evaluation for symptoms noted above with a computed tomography angiogram (CTA). Results showed diffuse nodular opacities throughout the lungs bilaterally with areas of coalescence and no lymphovascular invasion (Fig. 1C, D). On admission, the differential included infectious versus inflammatory processes, interstitial lung disease, or lymphangitic carcinomatosis. The patient was considered high risk for fungal and atypical mycobacterial infections due to living in mid-west and thin body habitus. Vitals upon admission included a temperature of 37.6 °C (99.6 Fahrenheit), blood pressure of 138/92 mmHg, heart rate of 116, respiratory rate of 39, and oxygen saturation of 97%. Comprehensive metabolic panel was notable for a sodium of 134 mmol/L (N: 136–145), lactic acid of 2.0 mmol/L (N: 0.5–1.9), alanine transaminase (ALT) 54 U/L (N: less than 33), and alkaline phosphatase 140 U/L (N: 35–104). Further studies included a procalcitonin that was negative at less than 0.05 ng/mL, C-reactive protein (CRP) elevated at 5.3 mg/dL (N: 0.0–5.0), sedimentation rate (ESR) of 80 mm/h (N: 0–20), and complete blood count only notable for an elevated white count of 23.91 × 10^3^/uL (N: 4.0–11.0 × 10^3^) with 95% neutrophils. Urinalysis was negative and imaging was obtained as shown below (Fig. 1A).

**Fig. 2:**
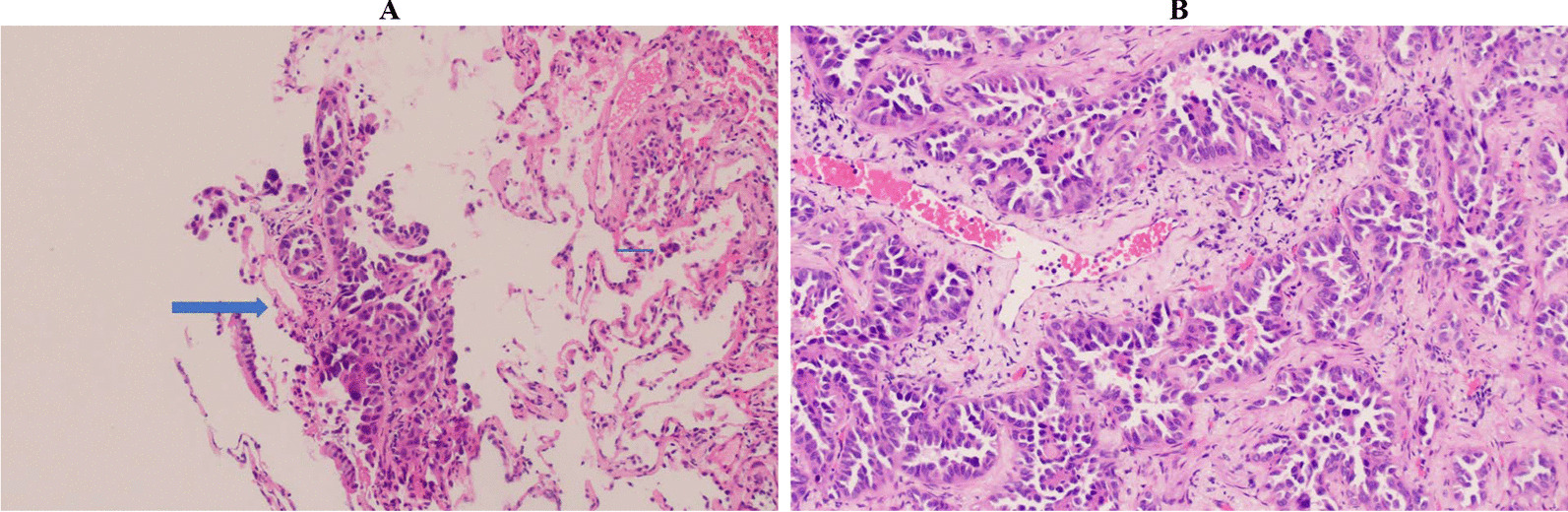
**A** 1/22 Pathology imaging at 20 × power from right middle lung bronchial biopsy showing lepidic adenocarcinoma, interpreted as primary lesion, with growth along intact alveolar septae. Big arrow points towards adenocarcinoma and little arrow points towards free floating tumor cells. **B** 1/29 Pathology imaging at 40 × power from right upper lobe wedge biopsy (3.4 cm and 1.9 cm at greatest length) confirming lepidic pattern adenocarcinoma, moderately differentiated with 2% PDL-1 expression. No regional lymph nodes were involved

The patient was admitted to the pulmonology service for thorough diagnostic evaluation and started on empiric antibiotics. Respiratory film array including SARS-CoV-2, urine streptococcus and legionella antigens, cryptosporidium, cytomegalovirus, histoplasma, aspergillus, herpes simplex virus, and clostridium difficile were performed and were all negative. The patient subsequently underwent a bronchoscopy with right middle lung biopsy and bronchial washings showing lepidic predominant adenocarcinoma, interpreted as primary lesion without stromal or lymphovascular invasion (Fig. 2A). Patient subsequently developed an iatrogenic right apical pneumothorax picked up on post procedure chest X-ray (CXR) showing a right pneumothorax with 3.2 cm pleural separation and prominent diffuse bilateral interstitial alveolar opacities (Fig. 1B). A 14 French right anterior chest tube was placed on suction. This was followed by a positron emission tomography (PET) scan showing diffuse hypermetabolic interstitial and airspace abnormality of the lungs without lymphadenopathy or distant metastatic lesions in addition to right hydropneumothorax and left pleural effusion (Fig. 1E, F).

Due to the patient’s atypical presentation and worsening hypoxemic respiratory failure requiring supplemental oxygen and persistent right apical pneumothorax, the patient underwent a video-assisted thoracoscopic surgery (VATS) along with talc pleurodesis and placement of a right apical and basilar chest tube. Surgical intervention included two right upper lobe wedge biopsies (3.4 cm and 1.9 cm at greatest length) and pleural peel biopsy (0.6 cm at greatest length) confirming lepidic predominant adenocarcinoma, moderately differentiated with positive parenchymal margins (invasion of visceral pleura). Programmed death ligand 1 (PDL-1) staining showed 2% expression. Advanced genomic testing showed a PTEN mutation.

Although the patient was being considered for trial of chemoimmunotherapy, the patient unfortunately perished from worsening respiratory failure on day 15 of hospitalization after family opted for comfort measures.

## Discussion and conclusions

Above we have reported a case of a healthy female succumbing to a rapidly deteriorating respiratory status secondary to bilateral lung involvement with diffuse pneumonic type lepidic adenocarcinoma (DP-LPA). LPA are being encountered frequently as having ground glass appearance on CT, are associated with increased resectability, favorable prognosis and overall diminished ability for nodal migration and extra-thoracic involvement [13–15]. LPA could present as solitary or multi-focal ground glass lesions [16]. Prognostically, favorable CT findings include small size, ground-glass opacities and air bronchograms, while unfavorable elements include spiculations, pleural retractions and size larger than 3.0 cm [17]. Studies have shown lepidic adenocarcinomas are low grade, acinar and papillary tumors are intermediate grade, and solid and micropapillary tumors are high grade [5, 18]. Lepidic adenocarcinomas have an average 5-year survival rate of 90% after surgical resection [19].

DP-LPA on the other hand is associated with worse prognosis but still maintains decreased predilection for nodal and distant metastases. Pneumonic-type involvement on imaging is defined as pneumonia like area of infiltrate or consolidation affecting a region of the lung; histologically these regions involve predominant lipidic growth pattern filling up portions of alveolar air spaces by mucin or tumor cells [20].

Similar to our case, multiple (US and non-US) population-based observational studies have shown that these multifocal LPA occur most commonly in middle age women, majority being non-smokers [20]. Radiographic studies commonly but not always correlate well with the histological findings and there also exists some interobserver variability among pathologists in identifying predominant pattern among lung adenocarcinomas [21]. Multifocal LPA can be mucinous, non-mucinous or mixed in nature. Whether DP-LPA is a different entity altogether or culmination of multifocal LPA process remains unclear. Consistent with our case about 60% of the patients with DP-LPA present with bilateral disease, generally with lack of nodal and distant metastases (Figs. 1, 2). In certain circumstances this had led to utilization of double lung transplantation process. However, most patients would eventually suffer recurrence in the transplanted organ.

Based on TNM staging our patient would be designated as T4 due to involvement of different lobes in one lung and M1a on accounts of contralateral lung/pleural involvement. Captured below in Table 1 are additional cases like ours representing scenarios of bilateral aerogenous-limited metastasis of lepidic adenocarcinoma. Such cases of DP-LPA have been associated by rapidly progressive acute respiratory failure with ARDS and succumb prior to receiving cancer directed therapy [22–26].

**Table 1:** Literature review: cases of lepidic predominant adenocarcinoma presenting as bilateral lung, micronodular infiltrates

Authors	Sex/age	Associated medical history	Positive diagnostic workup	Diagnostic imaging findings	Treatment
Azzeddine 2020	F/50	Personal: type II diabetes mellitus	Micro/Histo: invasive mucinous lepidic adenocarcinoma	CXR/CT: alveolar consolidation of left lower lobe with air bronchogram, multiple nodular lesions and alveolar condensation in right lung	Chemotherapy (names unspecified)
Daoud 2019	M/55	Personal: HTN, smoking (20-years), COPD, cocaine	Micro/Histo: aspergillus, HSV-1, pneumonic type adenocarcinoma	CXR/CT: diffuse bilateral upper/lower lobe opacities with nodular appearance, bilateral parenchymal infiltrates with mediastinal lymphadenopathy	Broad spectrum antibiotics (names not identified), voriconazole, acyclovir
Pathak 2019	F/60	Personal: smoking (20 years)	Micro/Histo: nonmucinous, lepidic predominant adenocarcinoma without invasion, positive TTF-1, EGFR, ALK, and PDL1 1%	CXR/CT: bilateral pulmonary infiltrates and ground glass opacities	Oncology treatment pursued (names unspecified)
Pathak 2019	M/55	Personal: GERD, HLD, smoking (20 years)	Micro/Histo: lepidic predominant adenocarcinoma	CXR/CT: bilateral ground glass opacities, greatest on the left	Oncology treatment pursued (names unspecified)
Jiménez-Zarazúa et al. (2018)	F/36	Personal: 33-weeks pregnant, smoking (5 years) Familial: type II diabetes mellitus, hypertension	Micro/Histo: moderately differentiated malignant neoplasia in lepidic pattern	CXR/CT: bilateral opacities, lower-lobe predominant	IV clarithromycin Death prior to cancer treatment
Mehic 2016	F/59	Personal: smoking (22 years), HTN	Micro/Histo: mucinous adenocarcinoma, lepidic predominant, KRAS positive, negative TTF1/napsin/CDX2/7/20	CXR/CT: reticular interstitial opacities with extended/deformed airways filled with mucous, bronchiectasis, thick interlobular septa	Antibiotics (unspecified), steroids Death prior to cancer treatment
Takanashi 2016	F/73	Personal: Not included in report	Micro/Histo: non-mucinous, lepidic-predominant invasive adenocarcinoma	CXR/CT: extensive ground glass opacities right lower lobe with infiltrative shadow	Pemetrexed Death prior to further treatment
Nguyen 2014	F/26	Personal: uncontrolled type II diabetes mellitus	Micro/Histo: lepidic predominant adenocarcinoma, mucin-secreting neoplastic cells and dense aggregates of mucinous debris in alveoli; positive CAM5.2 immunomarker	CXR/CT: dense perihilar opacities, areas of consolidation, ground glass infiltrates and cystic spaces bilaterally	IV antibiotics (names unspecified) Death prior to cancer treatment
Thimmareddygari 2021	M/47	Personal: Schizophrenia	Micro/Histo: invasive adenocarcinoma; positive for CK7, CK5/6, p63, and Napsin-A	CXR/CT: extensive bilateral airspace opacities, small bilateral pleural effusions, and scattered lucencies in several thoracic vertebrae	Death prior to cancer treatment (planned treatment with osimertinib)
Ismail 2017	F/53	Personal: type II diabetes and hypertension	Micro/Histo: acinar pattern adenocarcinoma positive for transcription termination factor and RNA polymerase 1	CXR/CT: diffuse bilateral infiltrates, diffuse bilateral confluent nodular and airspace opacities with areas of consolidation in both lung fields with focal mass like consolidation in left upper lobe and several mediastinal lymph nodes	No treatment specified in paper
Ismail 2017	F/36	Personal: no reported past medical history	Micro/Histo: micropapillary adenocarcinoma positive for TTF-1 and cytokeratin. FISH positive for rearrangement involving ROS1 gene	CXR/CT: diffuse bilateral interstitial infiltrates most notable in the upper lungs intermixed with ground glass infiltrates	No treatment specified in paper
Chang 2004	N/A/54	Personal: not included in report	Micro/Histo: adenocarcinoma	CXR/CT: diffuse patchy infiltration and areas of ground glass attenuation	No treatment specified in paper

Abbreviations include labs, short for laboratory workup, micro and histo, short for microscopic and histological evaluation, as well as *CXR* chest X-ray, *CT* computerized tomography, *IV* intravenous. Additional abbreviations include *HTN* hypertension, *COPD* chronic obstructive pulmonary disease, *GERD* gastroesophageal reflux disease, *HLD* hyperlipidemia

We outline a case of bronchoscopy and VATS-diagnosed lepidic adenocarcinoma with aerogenous spread in an otherwise healthy 63-year old Caucasian female. Admission evaluation and workup was notable for 2 months of progressive dyspnea, fatigue, and 15 pound weight loss. Laboratory workup was largely unremarkable apart from mild hyponatremia, elevated CRP, ESR, ALT and alkaline phosphatase. Several imaging studies throughout the patient’s hospital course identified bilateral lung involvement of diffuse micronodular opacities. All bacterial and fungal testing was negative and no improvement in constitutional symptoms were observed with a course of piperacillin-tazobactam, trimethoprim-sulfamethoxazole, or 9 days of oral steroids in a 40–20 mg taper. Because typical lepidic adenocarcinoma follows the path of the alveoli and bilateral aerogenous limited metastasis have been associated with rapidly progressive acute respiratory failure with ARDS, this case highlights the unique constitution adenocarcinomas can assume, even in a healthy, immunocompetent patient. This case report is aimed to help broaden the knowledge base and treatment protocols of DP-LPA.

## Data Availability

The data that support the findings of this study are available from EPIC but restrictions apply to the availability of these data, which is protected by HIPAA. For access to raw data, please contact Dr. Zaheer Akhtar, DO at zaheerakhtarmed@gmail.com.

## Abbreviations

NSCLC: Non-small cell lung cancer
SCLC: Small cell lung cancer
WHO: World Health Organization
CT: Computed tomography
CTA: Computed tomography angiogram
PET: Positron emission tomography
PDL-1: Programmed death ligand
KRAS: Kirsten rat sarcoma gene
ALK: Anaplastic lymphoma kinase gene
EGFR: Epidermal growth factor receptor
LPA: Lepidic predominant adenocarcinoma
CXR: Chest x-ray
VATS: Video assisted thoracoscopic surgery
DP-LPA: Diffuse pneumonic type lepidic adenocarcinoma
